# Gut bacteria-derived peptidoglycan induces a metabolic syndrome-like phenotype via NF-κB-dependent insulin/PI3K signaling reduction in *Drosophila* renal system

**DOI:** 10.1038/s41598-020-70455-7

**Published:** 2020-08-24

**Authors:** Olivier Zugasti, Raphäel Tavignot, Julien Royet

**Affiliations:** 1 Aix-Marseille Université, CNRS, IBDM, Marseille, France; 2 Institut Universitaire de France (IUF), Paris, France

**Keywords:** Chronic inflammation, Metabolic diseases

## Abstract

Although microbiome–host interactions are usual at steady state, gut microbiota dysbiosis can unbalance the physiological and behavioral parameters of the host, mostly via yet not understood mechanisms. Using the *Drosophila* model, we investigated the consequences of a gut chronic dysbiosis on the host physiology. Our results show that adult flies chronically infected with the non-pathogenic *Erwinia carotorova caotovora* bacteria displayed organ degeneration resembling wasting-like phenotypes reminiscent of Metabolic Syndrome associated pathologies. Genetic manipulations demonstrate that a local reduction of insulin signaling consecutive to a peptidoglycan-dependent NF-κB activation in the excretory system of the flies is responsible for several of the observed phenotypes. This work establishes a functional crosstalk between bacteria-derived peptidoglycan and the immune NF-κB cascade that contributes to the onset of metabolic disorders by reducing insulin signal transduction. Giving the high degree of evolutionary conservation of the mechanisms and pathways involved, this study is likely to provide a helpful model to elucidate the contribution of altered intestinal microbiota in triggering human chronic kidney diseases.

## Introduction

The human intestinal microbiota maintains a symbiotic relationship with its host under normal conditions. However, persistent modifications in the composition and/or in the function of this gut microbial population are associated with several pathologies, including metabolic diseases^1,2^. Among them, Metabolic Syndrome (MetS), a systemic disease also termed as dysmetabolic syndrome, is a world health concern thought to be caused by an underlying disorder of energy utilization and storage that directly increase the risk of diabetes mellitus type 2 as well as cardiovascular and renal diseases^3,4^. Although modifications in microbiota have been associated with MetS, it remains unclear whether the presence of keystone bacterial species in the gut is required for triggering the metabolic imbalance associated with the pathologies^5,6^. Increasing evidence however suggest that gut bacteria contribute to metabolic disturbances via the stimulation of low-grade inflammation of the gut and distal organs such as lung, brain, liver and kidney^2,7,8^. It is postulated that the translocation of gut bacterial pro-inflammatory metabolites and/or compounds to the circulation could trigger or at least facilitate systemic inflammation and mediate metabolic disorders leading to disease^9^. In most cases, the nature of the metabolites involved and their mode of action remain unknown.

Peptidoglycan (PGN) is a ubiquitous bacteria cell wall component for which the innate immune systems of plants, vertebrates and invertebrates have evolved various classes of sensing pattern recognition receptors among which NODs (Nucleotide Oligomerization Domains) and PGRPs (PeptidoGlycan‐Recognition Proteins) are the most studied^10–13^. Released by most dividing Gram-positive and Gram-negative gut bacteria, regardless of whether they are symbionts, pathogens, or pathobionts, PGN can be detected throughout the body suggesting a systemic diffusion from the gut microbiota^14–17^. In *Drosophila*, the direct binding of PGN to specific membrane-associated (PGRP-LC) or cytosolic (PGRP-LE) receptors triggers the production of hundreds of NF-κB-dependent immune effectors and regulators that could contribute consequently to inflammatory responses upon gut homeostatic imbalance such as alteration of microbiota^18–20^. A growing body of research in both invertebrate and vertebrate models continues to mount that microbiota-derived PGN could have systemic effects on metabolism and behaviors^21–23^. However, the causal relationships that underlie the systemic dissemination of PGN in promoting metabolic disorders and subsequent diseases are complex and not yet fully understood.

Studies on model organisms have provided valuable insights into genetic diseases in general and in the molecular mechanisms by which the gut microbiota is associated with innate immunity and metabolic disorders in particular^24–27^. Therefore, we used a *Drosophila* model of bacterial enteric dysbiosis to study the consequences of gut-derived PGN on the host’s physiology. A persistent dysbacteriosis can be established in flies by oral infection with the non-pathogenic Gram-negative enterobacteria *Erwinia caratovora caratovora* (*Ecc*)^14,28,29^. Sensing of *Ecc*-derived PGN triggers local and systemic activation of the IMmune Deficiency (IMD) signaling cascade, a NF-κB-dependent pathway that shares similarities with the mammalian Tumor-Necrosis-Factor Receptor1 (TNF-R1) pathway^30^. Combining phenotypic and comprehensive genetic analysis, we found that chronic systemic inflammation induced by gut-derived PGN causes a wasting syndrome whereby the abdomen becomes swollen due to fluid accumulation and the fat body and ovaries are almost degenerated^31,32^. We demonstrate that fluid buildup and fat body wasting are the direct consequence of a NF-κB-dependent local reduction of the insulin/PI3K signaling within the Malpighian tubules, the insect’s equivalent of kidneys. Altogether, this study provides support for the role of PGN as a specific bacteria-derived metabolite contributing to the onset of organ wasting and identifies the Malpighian tubules as major organs integrating immune and metabolic signals.

## Results

### Chronic enteric dysbiosis provokes a NF-κB-dependent fat body degeneration and bloating

**Figure 1:**
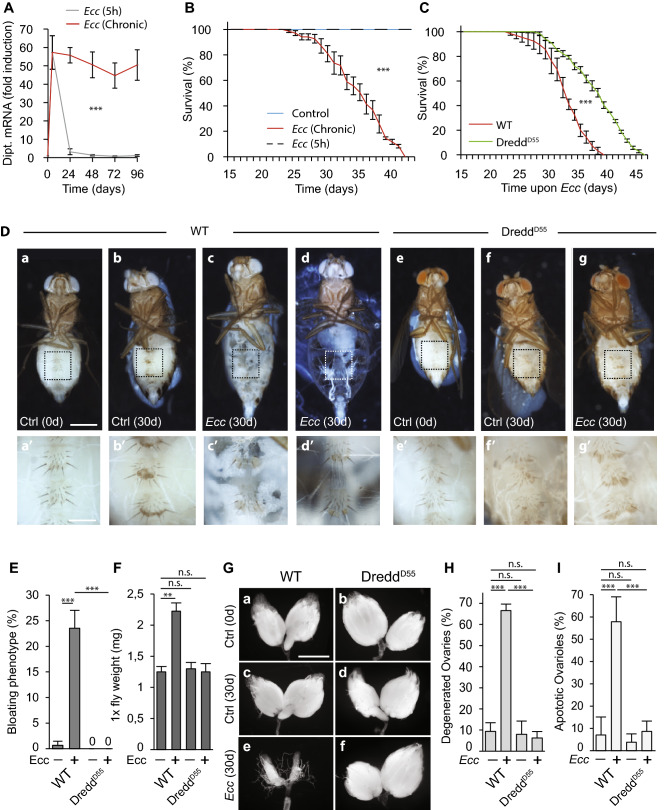
Chronic activation of the NF-κB cascade by bacteria-derived peptidoglycan reduces longevity and causes organ wasting. (**A**) Quantitative RT-PCR analysis of the expression of the NF-κB target gene *Diptericin* (Dipt.) in whole wild-type flies upon acute (5 h) and chronic enteric dysbiosis. The difference between control and chronically infected flies is significant (***p < 0.001; ANOVA test). (**B**) Survival of wild-type flies in control conditions and after acute (5 h) or chronic infection with *Ecc.* The difference between control and chronically infected flies is significant (***p < 0.001; one-sided log rank test). (**C**) Survival of wild-type and *Dredd*^*D55*^ mutant flies upon chronic infection. The difference between infected wild-type and *Dredd*^*D55*^ mutant flies is significant (***p < 0.001; one-sided log rank test). (**D**) Representative pictures of wild type (a–c) and *Dredd*^*D55*^ mutant flies (e–g) in control conditions (a, b and e, f) and infected with *Ecc* for 30 days (c, d and g) (scale bar, 0.5 mm). aʹ–gʹ, magnified fat bodies views of the boxed regions (scale bar, 0.15 mm). (**E**) Average percent of wild type and *Dredd*^*D55*^ mutant flies displaying abdominal bloating in control conditions or upon chronic infection. Comparisons between selected conditions are shown (Fisher’s exact test; ns, not significant; ***p < 0.001). (**F**) Weight measurements of wild-type and *Dredd*^*D55*^ mutant flies in control conditions or infected. Comparisons between selected conditions are shown (Mann Whitney test; ns, not significant; **p < 0.01). (**G**) Representative pictures of ovaries from wild-type and *Dredd*^*D55*^ mutant flies in control conditions (a, c and b, d, respectively) and after chronic dysbiosis (30 days) (e and f) (scale bar, 0.5 mm). (**H**) Average percent of ovary atrophy in wild-type and *Dredd*^*D55*^ flies in control conditions or upon enteric dysbiosis for 30 days. Comparisons between selected conditions are shown (Fisher’s exact test; ns, not significant; ***p < 0.001). (**I**) Average percent of ovarioles that contained apoptotic nurse cells in wild type and *Dredd*^*D55*^ flies in control conditions or infected for 30 days. Comparisons between selected conditions are shown (Mann Whitney test; ns, not significant; ***p < 0.001).

To evaluate the impact of chronic enteric dysbiosis on fly homeostasis, wild-type flies were contaminated every two days with a fresh solution of *Ecc*^14^. This entomopathogenic Gram-negative bacteria has been shown to release its cell-wall PGN which represents a strong elicitor of the innate immune signaling cascades in both flies and mammals^11,33^. In orally infected flies, gut-born bacteria PGN acts both at a short-range by triggering the IMD/NF-κB pathway in the surrounding enterocytes but also systemically in the whole animal after having crossed the gut epithelium and diffused into the hemolymph^14,15^. Consistently, while a short exposure to *Ecc* led to a rapid and transient activation of the IMD pathway (monitored through the transcription of one of its targets, the antimicrobial peptide gene *Diptericin*) without consequences on lifespan, persistent recolonization of the gastrointestinal tract with *Ecc* provoked a constant systemic activation of the NF-κB pathway and a premature death of the flies (Fig. 1A,B). From previous work, we expected that *Ecc*-dependent reduced life span was a direct consequence of PGN-mediated NF-κB pathway activation^34,35^. Indeed, lethality was worsened in absence of PGRP-LB, an amidase that reduces the intensity of the immune response by specifically degrading PGN, and partially rescued by genetically blocking the NF-κB cascade via the *Dredd* mutation (Fig. 1C and Sup Fig. 1). In order to evaluate the physiological consequences associated with PGN-dependent chronic NF-κB activation, we assessed putative internal organ damages. We observed that the fat body, an organ that preserves energy in the form of lipid droplets displayed very strong infection-dependent phenotypes. While fat body cells of non-infected flies covered the entire internal cuticle surface, the same tissue in chronically infected flies presented evident signs of degeneration with parts of the abdomen cuticle no longer covered by fat body cells (Fig. 1D and Sup Fig. 2A). Consistent with an infection-dependent wasting of the adipose tissue, a strong reduction of lipid droplets, the main organelle of fat storage, was observed in *Ecc* chronically fed flies, as compared to controls (Sup Fig. 2B). Moreover, almost 25% of chronically infected animals displayed a completely degenerated fat body and developed a severe “bloating phenotype” characterized by translucent and distended abdomens associated with a permanently extended proboscis (Fig. 1D,E, Sup Fig. 3A). We hypothesized that this abdominal bloating could arise from the accumulation of internal gas or fluid retention. Piercing inflated flies led to abdominal deflation without gas bubbles (data not shown). In addition, weight measurements revealed that chronically infected flies were almost twice as heavy as controls (Fig. 1F). Taken together, these results indicate that abdominal swelling is due to fluid accumulation. Furthermore, oral infection provoked an almost complete degeneration of ovaries associated with a massive increase in the percentage of apoptotic ovarioles (Fig. 1G–I and Sup Fig. 3B). The penetrance and the severity of all these phenotypes were decreased in the *Dredd* mutant background demonstrating that they were indeed the consequences of IMD pathway activation (Fig. 1D–I and Sup 2B). Altogether, our results demonstrate that a continuous activation of NF-κB signaling by bacteria-derived PGN induces fluid accumulation as well as fat body and ovary wasting, a systemic phenotype relevant to the wasting syndrome caused by metabolic disorders and reminiscent of MetS in mammals^31,32^.

### Chronic NF-κB signaling in enterocytes or adipocytes does not cause a wasting syndrome

**Figure 2:**
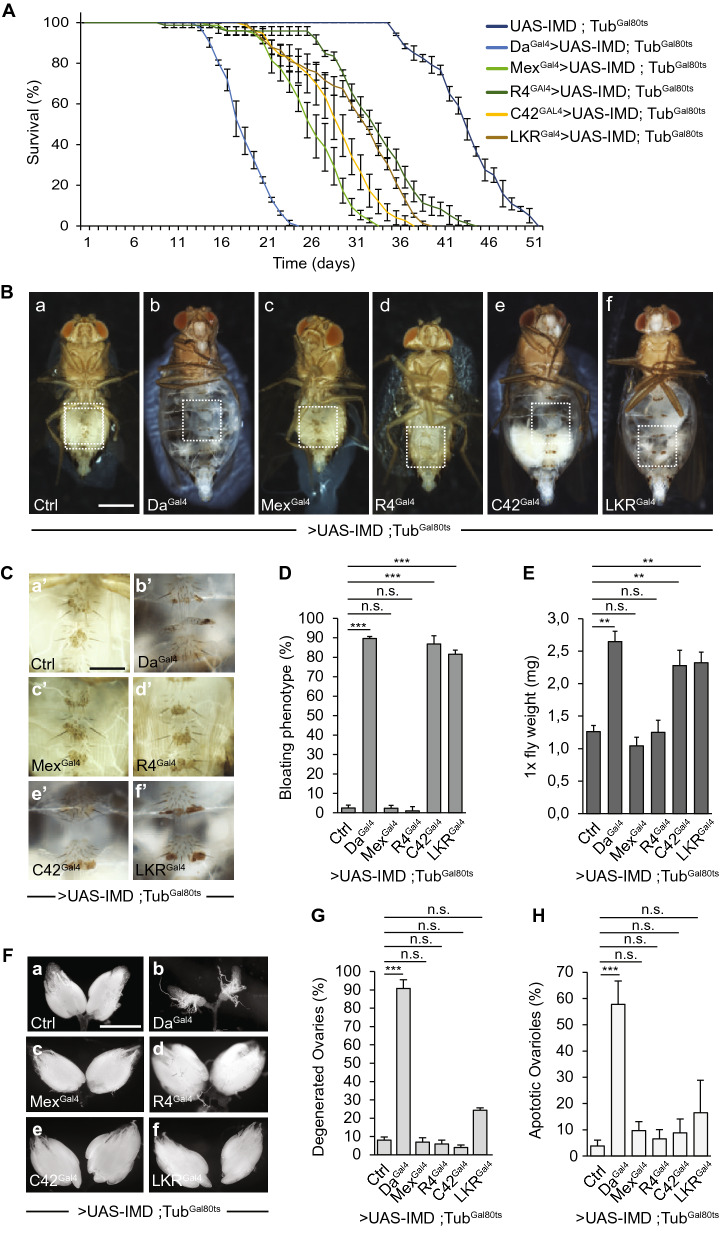
Constitutive IMD/NF-κB signaling in Malpighian tubule cells causes premature death, fluid buildup and fat body wasting. (**A**) Survival of control flies (UAS-IMD, Tub^Gal80ts^) and flies overexpressing IMD ubiquitously (Da^Gal4^ > UAS-IMD, Tub^Gal80ts^) or specifically in enterocytes (Mex^Gal4^ > UAS-IMD, Tub^Gal80ts^), fat body cells (R4^Gal4^ > UAS-IMD, Tub^Gal80ts^) and in Malpighian principal or stellate cells (C42^gal4^ > UAS-IMD, Tub^Gal80ts^ and LKR^Gal4^ > UAS-IMD, Tub^Gal80ts^, respectively). The difference between control flies and those overexpressing IMD is significant (p < 0.001 respectively; one-sided log rank test). (**B**) Representative pictures of control flies (a) and those overexpressing IMD (b–f) (scale bar, 0.5 mm). (**C**) Magnified fat bodies views of the boxed regions in (B) (scale bar, 0.15 mm). (**D**) Average percent of flies displaying abdominal bloating in control conditions and after overexpressing IMD. Comparisons between selected conditions are shown (Fisher’s exact test; ns, not significant; ***p < 0.001). (**E**) Weight measurements of control flies and those overexpressing IMD. Comparisons between selected conditions are shown (Mann Whitney test; ns, not significant; **p < 0.01). (**F**) Representative pictures of ovaries from control flies (a) and upon activation of the IMD cascade by overexpression of IMD (b–f) (scale bar, 0.5 mm). (**G**) Average percent of ovary atrophy in control flies and those overexpressing IMD. Comparisons between selected conditions are shown (Fisher’s exact test; ns, not significant; ***p < 0.001). (**H**) Average percent of ovarioles that contained apoptotic nurse cells in control conditions and upon constitutive activation of the IMD cascade*.* Comparisons between selected conditions are shown (Mann Whitney test; ns, not significant; ***p < 0.001).

Bacterial metabolites, other than PGN, can influence the host homeostasis^9^. For example, by producing Uracil, *Ecc* is inducing NADPH oxidase-dependent ROS production in the gut^36^. To probe the specific role of the PGN-dependent IMD activation in the apparition of a wasting syndrome, we tested whether activation of NF-κB signaling in the absence of bacteria could provoke phenotypes observed upon *Ecc* infection. By combining the binary system Gal4/UAS and the temperature-sensitive repressor Gal80^ts^, we observed that activation of the NF-κB cascade in adult tissues, via ubiquitous overexpression of IMD using the Da^Gal4^ driver, was sufficient to recapitulate all *Ecc*-triggered phenotypes confirming that they are the result of chronic activation of the immune system (Fig. 2, Sup Fig. 2C and Sup Fig. 4). Upon enteric dysbiosis, *Ecc-*derived PGN first triggers local activation of NF-κB signaling in enterocytes before activating a systemic immune response in fat body cells^14,15^. Constant activation of NF-κB signaling in enterocytes by overexpressing IMD using the Mex^Gal4^ driver (Sup Fig. 4) impacted fly lifespan but did not provoke fluid accumulation and had no obvious effect on fat body or ovary integrity (Fig. 2). Since the fat body is a multifunctional tissue implicated in energy storage, immune response and a major endocrine organ that secretes proteins governing oocyte maturation, we then tested whether constant NF-κB signaling in this tissue could recapitulate the phenotypes associated with *Ecc* chronic infection. To our surprise, although IMD/NF-κB pathway activation by overexpressing IMD in fat body cells using the R4^Gal4^ driver (Sup Fig. 4) significantly reduced fly lifespan, it did not caused abdominal bloating and had no obvious impact on the integrity of the fat body itself or on the ovaries (Fig. 2). Identical results were obtained when the IMD cascade was activated in the enterocytes or in the fat body cells of adult flies via the overexpression of the PGRP-LCa receptor (Sup Fig. 5)^37^. While chronic activation the IMD cascade in enterocytes or fat body cells reduces lifespan and might be involved in the premature death caused by *Ecc* chronic infection, the absence of degenerative phenotypes could reflect the need for synergistic activity of IMD activation in different tissues. Alternatively, it could also suggest that *Ecc*-dependent homeostasis disruption is the consequence of IMD pathway activation in PGN responding tissues other than the gut and the fat body and yet to be identified.

### NF-κB activation in Malpighian tubules mediates *Ecc*-induced fluid accumulation and fat body wasting

Abdominal swelling due to accumulation of fluid has been previously shown to be symptomatic of a failure of Malpighian tubules (MTs), an organ highly specialized in organic solute transport, metabolism and detoxification^38,39^. As MTs also constitute an autonomous immune-sensing tissue^20,40^, we tested the consequences of chronic NF-κB activation in the Malpighian tubule cells in the absence of infection. *Drosophila* Malpighian tubules are made of four tubules that consist of two cell types and ∼150 cells. Type I cells, also known as principal cells, through which cations and organic solutes are transported, are the major tubule cell type (~ 80%). Type II cells, also called stellate cells, through which water and chloride ion flow, are interspersed at regular intervals with the type I cells^41^. Interestingly, activating the NF-κB pathway in adult flies by overexpressing IMD or PGRP-LCa in either cell type using LKR^Gal4^ driver for stellate cells and C42^Gal4^ driver for principal cells (Sup Fig. 4) provoked premature death of the flies and recapitulated *Ecc*-triggered fluid retention and fat body wasting but not ovary degeneration (Fig. 2 and Sup Fig. 5). These results were confirmed using two independent drivers, Uro^Gal4^ for the principal cells and Tret-1^Gal4^ for the stellate cells (Sup Figs. 4 and 6). Simultaneous activation of the NF-κB pathway in principal and stellate cells strongly reduced fly longevity and accelerated the onset of the abdominal swelling and fat body depletion phenotypes without affecting ovary integrity (Sup Fig. 7). Collectively, these results suggest that activation of the NF-κB pathway in either cell type of Malpighian tubules can act in parallel to cause death by inducing progressive deterioration of MTs function and distant fat body wasting but preclude a direct involvement in the ovary degeneration caused by enteric dysbiosis.

### Intracellular detection of PGN by MT cells activates NF-κB signaling and causes fluid retention and fat body wasting

**Figure 3:**
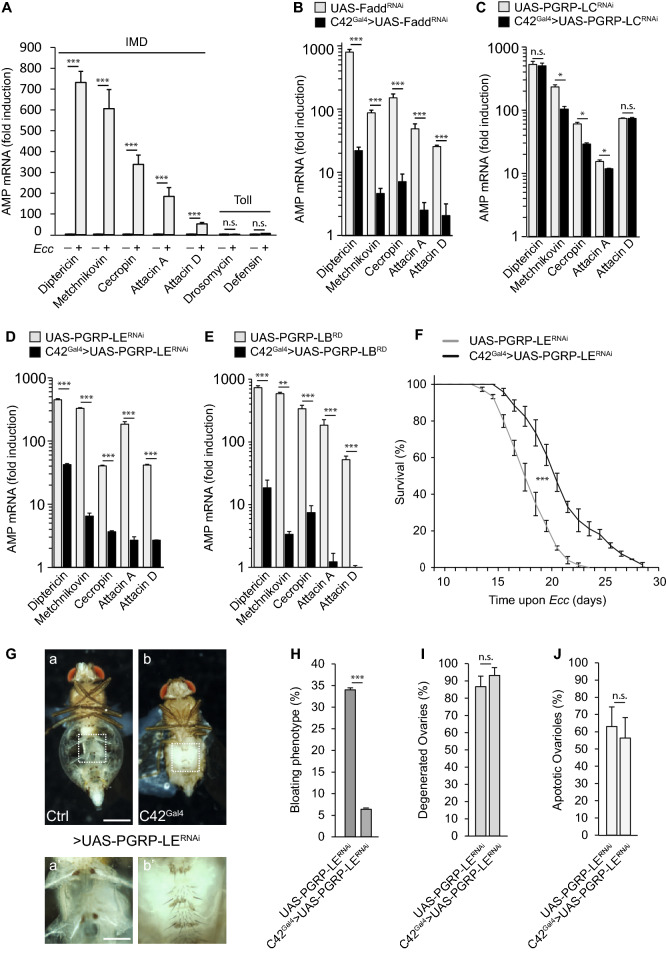
Intracellular detection of PGN by MT cells activates NF-κB signaling and causes fluid retention and fat body degeneration. (**A**) Quantitative RT-PCR analysis of the expression of IMD/NF-kB target AMP genes Diptericin, Metchnikovin, Cecropin, Attacin A and Attacin D and Toll target genes Drosomycin and Defensin in Malpighian tubules upon enteric dysbisosis. Comparisons between control and *Ecc* infected flies are shown (Mann Whitney test; ns, not significant, ***p < 0.001). In this and subsequent RT-PCR assays, the analysis of the activation of the NF-κB signaling was performed 24 h after oral infection with *Ecc*. (**B**–**E**) Quantitative RT-PCR analysis of the expression of IMD/NF-κB antimicrobial target genes upon enteric dysbiosis after RNAi-mediated inactivation of NF-κB pathway components Fadd (**B**), PGRP-LC (**C**) and PGRP-LE (**D**) or overexpression of the cytosolic amidase PGRP-LB^RD^ (**E**) in MT principal cells. Results are presented relative to those of control uninfected flies. Comparisons between selected conditions are shown (Mann Whitney test; ns, not significant, ***p < 0.001, **p < 0.01; *p < 0.1). (**F**) Survival of control flies (UAS-PGRP-LE^RNAi^) and after RNAi-mediated inactivation of PGRP-LE in MT principal cells (C42^Gal4^ > UAS-PGRP-LE^RNAi^) upon chronic enteric dysbiosis. The difference between these conditions is significant (p < 0.001; one-sided log rank test). (**G**) Representative pictures of control infected flies (a) and after PGRP-LE RNAi-mediated gene knock-down in principal cells (b) (scale bar, 0.5 mm). aʹ and bʹ, magnified fat bodies views of the boxed regions (scale bar, 0.15 mm). (**H**–**J**) Quantification of abdominal bloating (H), ovary degeneration (**I**) and ovarioles that contained apoptotic nurse cells (**J**) upon chronic infection in control flies and after PGRP-LE RNAi-mediated gene knock-down in principal cells. Comparisons between selected conditions are shown (Fisher’s exact test (**H**,**I**) and Mann Whitney test (**J**); ns, not significant, ***, p < 0.001).

To confirm that NF-κB pathway activation in the MTs is the trigger of *Ecc*-mediated fluid accumulation and fat body degeneration, we analyzed the immune status of the MTs of *Ecc*-orally infected flies. Antimicrobial peptide (AMP) targets of the IMD/NF-κB pathway but not of the Toll/NF-κB pathway, were highly induced in MTs 24 h after *Ecc* oral infection (Fig. 3A). This activation was almost fully suppressed when the IMD pathway adaptor Fadd was specifically inactivated via RNAi in principal cells suggesting that these cells ensure the main production of antimicrobial peptides in response to PGN during enteric dysbiosis (Fig. 3B). In addition, elimination of the cytosolic PGN receptor PGRP-LE, but not the transmembrane receptor PGRP-LC, was also sufficient to abolish the *Ecc-*dependent activation of AMP genes in MTs suggesting an intracellular detection of PGN (Fig. 3C,D)^18,20^. These results were confirmed using the cytosolic PGN-degrading enzyme, PGRP-LB^RD^^15^. Specific expression of PGRP-LB^RD^ in principal cells completely prevented IMD/NF-κB AMP targets induction in adult flies 24 h after *Ecc* feeding (Fig. 3E). Consistently, reducing IMD pathway activation either via RNAi-mediated inactivation of PGRP-LE, or via PGRP-LB^RD^ overexpression, was sufficient to partially rescue the *Ecc*-triggered lethality and to reduce the penetrance of the bloating phenotype and fat body wasting without bettering the ovary atrophy (Fig. 3F,J and Sup Fig. 8). Altogether, these data demonstrate that, by activating NF-κB within Malpighian tubule cells, gut-born *Ecc* PGN contributes to infection-induced fluid accumulation and fat body wasting.

### Chronic activation of NF-κB interrupts insulin/PI3K signaling in MTs

**Figure 4:**
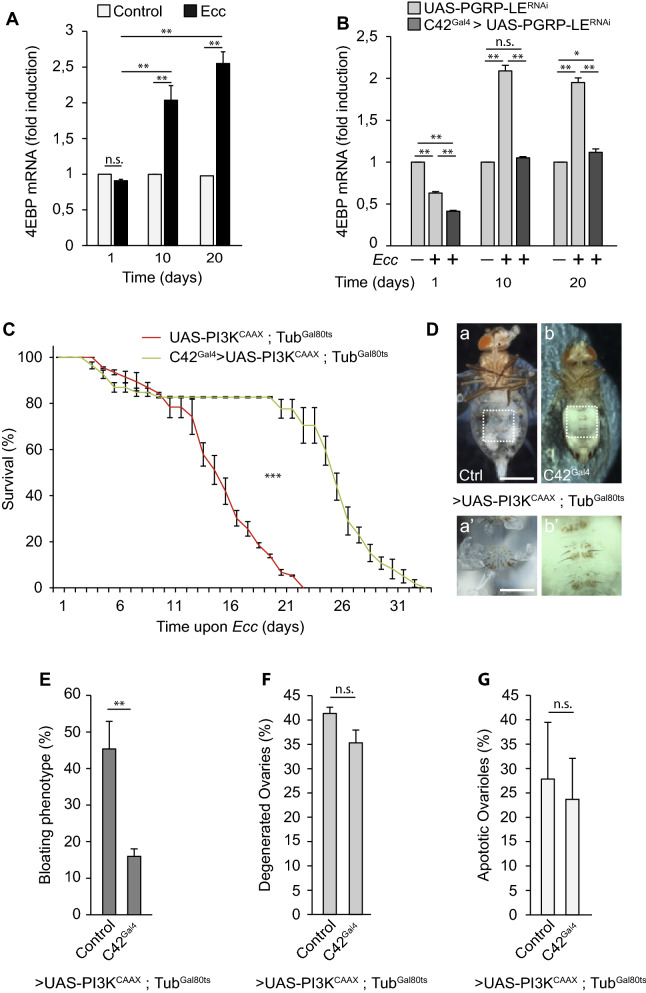
Chronic activation of the IMD/NF-κB pathway in MTs interrupts insulin signaling and causes fluid accumulation and fat body wasting. (**A**) Quantitative RT-PCR analysis of the expression of the FoxO target gene 4EBP in Malpighian tubules of control and infected flies for 1, 10 and 20 days. Comparisons between selected conditions are shown (Fisher’s exact test; ns, not significant; **p < 0.01). Each condition was normalized to its own uninfected control to take into account age-specific changes in gene expression. (**B**) Quantitative RT-PCR analysis of the expression of 4EBP in Malpighian tubules of control and infected flies for 1, 10 and 20 days treated or not with RNAi against PGRP-LE in principal cells**.** Comparisons between selected conditions are shown (Fisher’s exact test; ns, not significant; **p < 0.01, *p < 0.1). (**C**) Survival of infected control flies (UAS-PI3K^CAAX^, Tub^Gal80ts^) and those overexpressing a constitutive active form of PI3K in Malpighian principal cells (C42^gal4^ > UAS-PI3K^CAAX^, Tub^Gal80ts^). The difference between control flies and flies overexpressing PI3K^CAAX^ in MT principal cells is significant (***p < 0.001, respectively; one-sided log rank test). (**D**) Representative pictures of infected control flies (a) and those overexpressing PI3K^CAAX^ under the control of the C42^gal4^ driver (b) (scale bar, 0.5 mm). aʹ and bʹ, magnified fat bodies views of the boxed regions (scale bar, 0.15 mm). (**E**–**G**) Percentage of abdominal bloating (**E**), ovary degeneration (**F**) and ovarioles that contained apoptotic nurse cells (**G**) upon chronic infection of control flies and those overexpressing PI3K^CAAX^ in MT principal cells. Comparisons between selected conditions are shown (Fisher’s exact test (**E**,**F**) and Mann Whitney (**G**) test; ns, not significant, **p < 0.01).

Reduction of the metabolic insulin/PI3K signaling cascade is a central feature to development of metabolic disturbances in mammals in general and in *Drosophila* in particular^20,31,42^. We therefore asked whether the effects of PGN on MTs could be mediated by a local inhibition of this pathway. We first tested the consequences of gut chronic infection on the well-characterized insulin/PI3K target gene 4EBP which transcription is increased when insulin signaling is antagonized^31,43^. Chronical *Ecc* feeding was sufficient to increase 4EBP transcription in MTs, a feature of a local PI3K/insulin signaling downregulation (Fig. 4A). This activation was fully abrogated when either PGRP-LE or Fadd were silenced via RNAi (Fig. 4B and Sup Fig. 9A) or after the overexpression of PGRP-LB^RD^ in the principal cells of *Ecc*-infected flies (Sup Fig. 9B). To assess whether IMD-dependent insulin signaling decrease in MTs was involved in the different phenotypes associated with the wasting syndrome, we probe whether expressing a constitutive active form of PI3K (PI3K^CAAX^) in MT principal cells could rescue the *Ecc*-mediated phenotypes. We observed that the *Ecc*-induced lethality, the bloating phenotype and the fat body wasting but not the degeneration of ovaries were partially rescued by restoring the PI3K cascade in MT principal cells (Fig. 4C–G). Altogether, these results show that chronic activation of the IMD pathway by bacteria-derived PGN leads to a local reduction of the insulin/PI3K pathway in MTs causing fluid accumulation and fat body wasting.

### Impaired FoxO and TOR metabolic signaling in MTs provoke fluid buildup and fat body wasting

**Figure 5:**
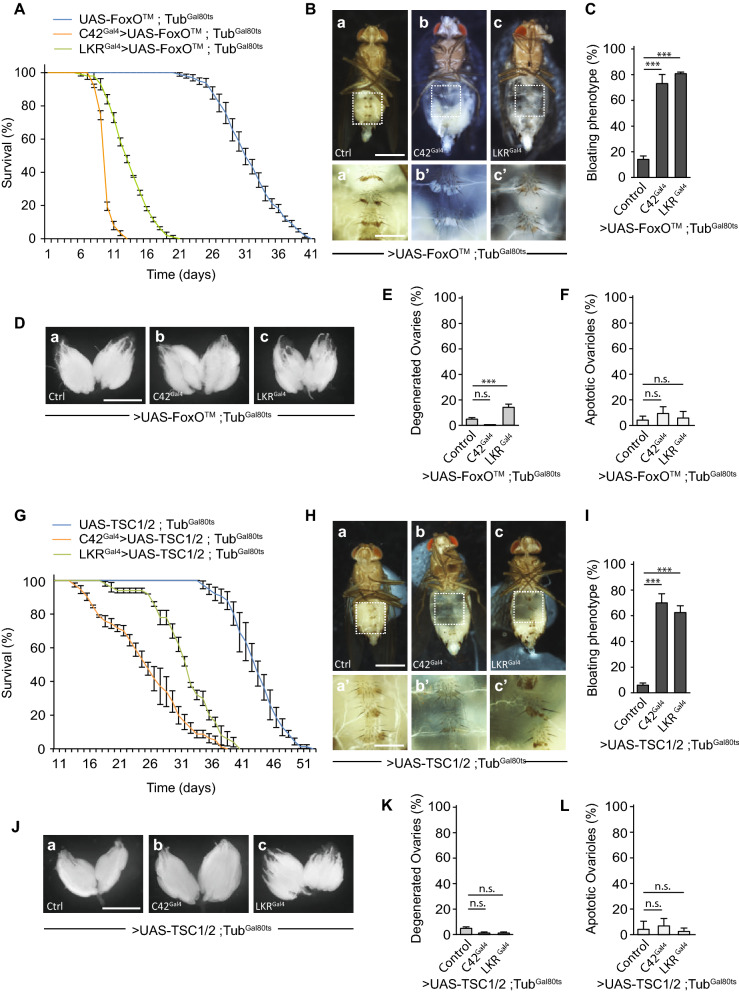
Activation of FoxO or inhibition of TOR signaling in MTs provokes fluid buildup and fat body wasting. (**A**) Survival of control flies (UAS-FoxO™, Tub^Gal80ts^) and flies expressing FoxO™ in Malpighian principal or stellate cells (C42^gal4^ > UAS-FoxO™, Tub^Gal80ts^ and LKR^Gal4^ > UAS-FoxO™, Tub^Gal80ts^, respectively). The difference between control flies and flies expressing FoxO™ is significant (p < 0.001 respectiveley; one-sided log rank test). (**B**) Representative pictures of control flies (a) and those expressing FoxO™ (b and c) (scale bar, 0.5 mm). aʹ–cʹ**,** magnified fat bodies views of the boxed regions (scale bar, 0.15 mm). (**C**) Average percent of abdominal bloating observed in flies expressing FoxO™. Comparisons between selected conditions are shown (Fisher’s exact test; ***p < 0.001). (**D**) Representative pictures of ovaries from control flies (a) and upon expression of FoxO™ (b and c) (scale bar, 0.5 mm). (**E**,**F**) Average percent of ovary atrophy (E) and ovarioles that contained apoptotic nurse cells (F) in control flies and those expressing FoxO™. Comparisons between selected conditions are shown (Fisher’s exact test (E) and Mann Whitney test (F); ns, not significant, ***p < 0.001). (**G**) Survival of control flies (UAS-TSC1/2, Tub^Gal80ts^) and flies overexpressing TSC1/2 in Malpighian principal or stellate cells (C42^gal4^ > UAS-TSC1/2, Tub^Gal80ts^ and LKR^Gal4^ > UAS-TSC1/2, Tub^Gal80ts^, respectively). The difference between control flies and flies overexpressing TSC1/2 is significant (p < 0.001 respectively; one-sided log rank test). (**H**) Representative pictures of control flies (a) and those overexpressing TSC1/2 (b and c) (scale bar, 0.5 mm). aʹ–cʹ, magnified fat bodies views of the boxed regions (scale bar, 0.15 mm). (**I**) Average percent of abdominal bloating observed in flies overexpressing TSC1/2. Comparisons between selected conditions are shown (Fisher’s exact test; ***p < 0.001). (**J**) Representative pictures of ovaries from control flies (a) and upon inhibition of the TOR signaling transduction cascade by overexpression of TSC1/2 (b and c) (scale bar, 0.5 mm). (**K**,**L**) Average percent of ovary atrophy (**K**) and ovarioles that contained apoptotic nurse cells (L) in control flies and flies overexpressing TSC1/2. Comparisons between selected conditions are shown (Fisher’s exact test, (**K**) and Mann Whitney test (**L**); ns, not significant).

The evolutionary conserved transcription factor FoxO (Forkhead box O) and TOR (Target of Rapamycin) complex mediate most of the PI3K/insulin signaling metabolic effects^44,45^. These key effectors are involved in the integration of environmental cues and their translation into metabolic responses^46^. Alterations in insulin signaling result in both FoxO nuclear translocation and consequent transcription of target genes and inhibition of the TOR complex. We, therefore, tested whether either FoxO activation or TOR inhibition in MTs was sufficient to recapitulate the consequences of PI3K/insulin signaling reduction in the excretory system during enteric dysbiosis. Indeed, specific expression of a constitutive active form of FoxO (FoxO™)^43^ either in the Malpighian principal or stellate cells in non-infected flies reduced life span and provoked a bloating phenotype associated with fat body depletion but did not affect the integrity of ovaries (Fig. 5A–F). Similarly, overexpression of the heterodimeric tuberous sclerosis complex TSC1/TSC2, the main inhibitor of TOR^47^, was sufficient to provoke abdominal swelling and fat body degeneration but had not impact on the integrity of ovaries (Fig. 5G–L). Altogether these results plead in favor of a model in which local reduction of PI3K/insulin signaling in MTs caused by the activation of NF-κB by gut-derived PGN alters FoxO and TOR metabolic signaling, and *in fine* provoke imbalance in fluid homeostasis and wasting of the fat body.

## Discussion

MetS, defined by a clustering of metabolic disorders affecting multiple interacting organ systems, is a worldwide health concern with high morbidity and mortality^3^. Recent studies in animal models and in humans support a link between persistent low-grade inflammation caused by microbiota-derived metabolites and various components of MetS-associated chronic diseases^5,48^. The identification of these metabolites and their mechanisms of action are therefore critical for uncovering the role exerted by bacterial ecosystems on the setting of metabolic diseases. This study establishes a clear crosstalk between bacteria-derived PGN and an immune NF-κB signaling that contributes to the onset of metabolic disorders in *Drosophila*. Adult flies chronically infected with the enterobacteria *Ecc* develop phenotypes that are associated with impaired metabolic homeostasis including fluid buildup as well as fat body and ovary degeneration^31,32^. We made the demonstration that systemic activation of the IMD/NF-κB cascade by gut-derived PGN is the main elicitor of these physio-pathologic consequences. Our data clearly indicate that upon chronic enteric dysbiosis, a specific NF-κB-dependent reduction of insulin/PI3K signaling in MTs is the trigger of fluid accumulation and fat body wasting but not ovary atrophy. It is noteworthy that interruption of systemic insulin signaling by ImpL2, which antagonizes insulin signaling by binding to *Drosophila* insulin-like peptides (DILPs), and bacteria-derived PGN cause a wasting syndrome affecting fluid homeostasis, fat body and ovaries^31,49^. Thus, it will be of future interest to determine whether chronic IMD pathway activation in PGN responding organs other than MTs also impact local or systemic insulin/PI3K signaling.

Previous reports have linked bacteria-immunity and insulin signaling in flies, although most studies have been performed at the larval stage. Persistent activation of IMD was shown to deplete fat reserves, cause hyperglycemia, delay development, and impair larval growth suggesting that IMD influences metabolic regulatory pathways^50^. Humoral infection with the bacterial pathogen *Mycobacterium marinum*, a close relative of *M. tuberculosis* causes a progressive loss of metabolic energy stores in form of fat and glycogen leading to a wasting-like phenotype. This wasting response is in part the consequence of inhibition of the insulin signaling and consequent activation of the transcription factor FoxO^51^. Finally, activation in the larval fat body of the Toll pathway, the other fly NF-κB signaling cascade, was shown to block insulin-dependent growth and nutrient storage. Since DILP6 is a selective target of Toll signaling, it was proposed that Toll signaling reduces growth by inducing hormone insufficiency in the case of infection^52^. Whereas most studies have identified the fat body as the main tissue integrating immune and metabolic signals, we showed that the wasting syndrome caused by bacteria-derived PGN is largely fat body independent. Indeed, it is in part the consequence of a PGN dependent insulin signaling reduction in the excretory system highlighting that Malpighian tubules are essential organs for the maintenance of immune and metabolic homeostasis in adult flies. It is interesting to note that PGN from Gram-positive bacteria, called Lys-type PGN is filtered by another component of the excretory system of the insects, the nephrocytes. In flies with no functional nephrocytes, Lys-type PGN accumulate in the hemolymph leading to Toll/NF-kB constitutive activation which is detrimental for the flies^53^.

How is IMD activation impairing the insulin pathway in the renal tubules and how the local reduction of this metabolic pathway impacts remote organs such as the fat body remain open questions. One model will involve a modulation of the levels of circulating DILPs. Indeed, although in adult flies the metabolic role of DILPs appears complex, a reduction of expression of these insulin analogs has been suggested to cause infection-induced wasting^51^. In MTs, DILP5 produced by principal cell acts as a local insulin agonist whose expression is modulated upon multiple stress conditions^54^. Therefore, during enteric dysbiosis, a NF-κB dependent reduction of DILP5 production or release by MTs could locally impair insulin signaling hence affecting renal functions. It could also perturb peripheral target organs for insulin including the fat body. Interestingly, DILP5 production by MTs is under the control of tachykinin, an endocrine peptide produced by the enteroendocrine gut cells in an IMD-dependent manner^54^.

Altogether, our results provide compelling evidence that bacteria-derived PGN triggers IMD/NF-κB pathway activation and metabolism disorders in MTs contributing to organ failure and distant fat storage wasting in flies. It is worth to note the remarkable parallels between the results obtained and the physiopathology of the human Chronic Kidney Disease (CKD), a MetS-associated chronic pathology. Mounting evidence show that alterations of gut microbiota play a key role in CKD and cause systemic NF-κB mediated inflammation and alteration of the insulin/PI3K metabolic signaling^55,56^. CDK is characterized by a slow and progressive loss of glomerular filtration rate leading to kidney failure and fluid retention^57^. The complications of this disease also impact beyond the kidney and CKD is associated with multiple physiological and metabolic disturbances including muscle and fat storage wasting^58^. Based on the relevance of *Drosophila* MTs as a model for human renal diseases and the conservation of immune–metabolic interactions from invertebrates to vertebrates^59^, it is tempting to propose that low grade inflammation caused by PGN contributes to the onset of metabolic diseases such as CKD. If so, circulating PGN could be used as an early diagnostic biomarker or a novel therapeutic target to prevent chronic inflammation associated with gut dysbiosis in MetS. The recent validation of the first blocking anti-PGN antibody is, in this respect, an important first step^57^.

## Methods

### *Drosophila melanogaster* strains and maintenance

The *w*^*1118*^ (BL #6326) flies was used as the reference control wild-type strain. The PGRP-LB^Δ^^34^ and Dredd^D55^^60^ mutant strains were generous gifts of B. Lemaitre. The following strains were obtained from the Bloomington Stock Center: Da^Gal4^ (BL#55851), R4^Gal4^ (BL#33832), C42^Gal4^ (BL#30835), Uro^Gal4^ (BL#44416), Tub^Gal80ts^ (BL#7018 and BL#7019), UAS-PI3K^CAAX^ (BL#8294), UAS-Relish^RNAi^ (BL#28943), UAS-PGRP-LE^RNAi^ (BL#60038) and UAS-mCD8RFP (BL#27398). The UAS-Fadd^RNAi^ and UAS-PGRP-LC^RNAi^ lines were kindly provided by Meier^61^, and Lemaitre^19^. The drivers LKR^Gal4^ and Tret1-1^Gal4^ were kindly provided by Al-Anzi^62^ and Schirmeier^63^, respectively. The UAS-PGRP-LB^RD^ strain was described elsewhere^63^. The UAS-FoxO™ was kindly provided to our laboratory by Tatar^43^. The fly stocks used for this study also include Mex^Gal4^^64^, UAS-PGRP-LCa^37^ and UAS-TSC1/2 strains^65^. Unless otherwise indicated, flies were grown at 25 °C on a yeast/cornmeal medium in 12 h/12 h light/dark cycle-controlled incubators. Flies of genotypes containing the Gal4/UAS/Gal80^ts^ constructs were reared at 20 °C and transferred to 29 °C as 5-day-old adults to allow the expression of the Gal4 transcription factor. The five days of adulthood prior to the onset of Gal4 expression are required to allow maturation of adult structures. Female flies were used for all experiments.

### *Drosophila* culture media

For 1liter of food, 8.2 g of agar (VWR, cat. #20768.361), 80 g of cornmeal flour (Westhove Maize H1) and 80 g of yeast extract (VWR, cat. #24979.413) were cooked for 10 min in boiling water. 5.2 g of Methylparaben sodium salt (Merck, cat. #106756) and 4 ml of 99% propionic acid (CARLO ERBA, cat. #409553) were added when the food had cooled down. For antibiotic (ATB) treatment, standard medium was supplemented with Ampicillin, Kanamycin, Tetracyclin and Erythromycin at 50 μg/ml final concentrations^15^.

### Infection of adult flies by *Ecc* and survival assays^15^

The bacterial strain used in this study is *Erwinia carotovora carotovora* 15 (*Ecc*) cultured in Luria–Bertani (LB) medium at 30 °C overnight. Bacterial cultures were centrifuged at 2,500*g* for 20 min at RT. Cells were serially diluted in PBS and their concentration was determined by optical density (OD) measurement at 600 nm. For oral infection and subsequent analysis, 5-day-old females raised from egg-to-adult in presence of ATB in the food were used. 24 h before the infection, flies were transferred in vials without ATB and then placed in a fly vial with *Ecc* contaminated food. This food solution was obtained by mixing a pellet of an overnight culture of bacteria *Ecc* (OD = 200) with a solution of 5% sucrose (50/50) and added to a filter disk that completely covered the agar surface of the fly vial. To perform survival assays upon chronic infection, flies were infected every 2 days with a fresh solution of *Ecc* (OD = 200) with a solution of 5% sucrose (50/50). For kinetics involving an acute exposure to *Ecc*, flies were in contact with infected food for 5 h before being transferred to vials containing a filter disk soaked in a food solution of LB medium with of 5% sucrose (50/50) in presence of ATB. Survival analysis in absence of infection were conducted in vials containing a food solution of LB medium and a solution of 5% sucrose (50/50) in presence of ATB. Dead flies were scored at a set time once a day. A minimum of three vials of 25 flies were used for each survival assay. Experiments were repeated at least twice^15^. Statistical analyses used one-sided log rank test within Prism (GraphPad software).

### Organ wasting analysis and ovarian health quantification

The penetrance of the abdominal bloating was analyzed during dedicated kinetics at a set time once a day until all flies were dead. In practice, flies were anesthetized with CO_2_ and observed individually under a stereo microscope. Those displaying a translucent and distended abdomen were scored and transferred to a different fly vial. At least three vials of 25 flies were used for each assay. Statistical analyses used the Fisher’s exact test within Prism (GraphPad software). For neutral lipid staining, carcass/fat body of control and infected flies were dissected in cold PBS, fixed for 30 min in 4% paraformaldehyde on ice, rinse 3 times in PBS and stained with BODIPY 493/503 at 1:1,000 in PBS for 30 min. Ovarian degeneration was determined after dissection by direct observation and quantification of the substantial reduction in ovarian size and loss of mature eggs at 50% of lethality. A minimum of 20 ovaries were analyzed in each assay. Experiments were conducted in triplicate. Statistical analyses used Mann–Whitney test within Prism (GraphPad software). Ovarian health was quantified as the percentage of ovarioles that contained one or more apoptotic egg chamber at stage 9–10^49^. Briefly, ovaries were dissected in PBS and fixed 30 min in 4% paraformaldehyde on ice. DAPI staining was used as a tool to visualize egg chambers throughout oogenesis and identify those that are dying during mid-oogenesis^66^. For each experimental condition, a minimum of one hundred ovarioles from at least 20 ovaries were analyzed on fluorescence microscope with UV. Statistical analyses used the Mann–Whitney test within Prism (GraphPad software).

### Fly weight measurements

To measure body weight, female flies (n = 24) were anaesthetized with CO_2_, transferred to Eppendorf tubes and weighed on a METTLER TOLEDO precision balance.

### Quantitative real-time PCR

RNA from whole adult animals or dissected organs (n = 15) was extracted with RNeasy Mini Kit (QIAGEN, cat. #74106). Quantitative real-time PCR, TaqMan, and SYBR Green analysis were performed as previously described^15^. The amount of mRNA detected was normalized to control rp49 mRNA values. Normalized data was used to quantify the relative levels of a given mRNA according to cycling threshold analysis (ΔCt). Control and experimental conditions were tested in the same ‘run’. Each sample was normalized to its own rp49 control to take into account age-specific changes in gene expression. Results are presented as average and standard deviation of a minimum of three independent experiments. Statistical analyses used Mann–Whitney test, within Prism (GraphPad software).

### qRT-PCR primers

Primers used for qRT-PCR are for:

- rp49: GACGCTTCAAGGGACAGTATCTG, AAACGCGGTTCTGCATGA
- Diptericin: GCTGCGCAATCGCTTCTACT, TGGTGGAGTGGGCTTCATG
- Metchnikovin: GATGCAACTTAATCTTGGAGCG, TTAATAAATTTGGACCCGGTCTTGGTTGG
- Cecropin: ATGAACTTCTACAACATCTTCG, GGCAGTTGCGGCGACATTGGCG
- Attacin A: CCCGGAGTGAAGGATG, GTTGCTGTGCGTCAAG
- Attacin D: GTCACTAGGGTTCCTCAG, GCCGAAATCGGACTTG
- Drosomycin: CGTGAGAACCTTTTCCAATATGATG, TTCCACGACCACCAGCAT
- Defensin: GTTCTTCGTTCTCGTGG, CTTTGAACCCCTTGGC
- 4EBP: CATGCAGCAACTGCCAAATC, CCGAGAGAACAAACAAGGTGG

### Gal4 expression profile imaging

Dissected organs (intestine, Malpighian tubules and carcass/fat body) from 4–6-day old female flies expressing the membrane tagged red fluorescent protein mCD8RFP under the control of the Gal4 drivers used in this work were fixed for 20 min in 4% paraformaldehyde on ice and rinse 3 times in PBT (PBS + 0.3% Triton X-100). DAPI staining was used as a tool to visualize nuclei. Tissues were then mounted in VECTASHIELD (Vector Laboratories) fluorescent mounting medium. Images were captured with a LSM 780 ZEISS confocal microscope and processed using Adobe Photoshop.

## Supplementary information

Supplementary Information 1.

Supplementary Information 2.
