# Supplementary material for: Gut bacteria-derived peptidoglycan induces a metabolic syndrome-like phenotype via NF-κB-dependent insulin/PI3K signaling reduction in Drosophila renal system
Source: Sci Rep. 2020 Aug 24;10:14097. doi: 10.1038/s41598-020-70455-7 (PMC7445169; doi:10.1038/s41598-020-70455-7)
Supplement: Supplementary file 1 — Supplementary Information 1. [file 41598_2020_70455_MOESM1_ESM.docx]

**SUPPLEMENTARY FIGURE LEGENDS**

**Sup Figure 1.** ***Ecc*-dependent reduced life span is a direct consequence of PGN-mediated NF-kB pathway activation.** Survival of wild type, PGRP-LB^Δ^ and Dredd^D55^; PGRP-LB^Δ^ mutant flies upon chronic oral infection. The difference between chronically infected PGRP-LB^Δ^ and Dredd^D55^; PGRP-LB^Δ^ animals is significant (***, p<0.001; one-sided log rank test).

**Sup Figure 2. Chronic activation of NF-kB cascade induces fat body wasting and lipid stores depletion. A.** Representative stereo microscopic pictures of transgenic flies expressing the red fluorescent protein (DsRed) in fat body cells in control conditions (a) and infected with *Ecc* for 10 days (e) (scale bar, 0.5 mm). Magnified stereo microscopic, fluorescent (DsRed) and merge views (b-d and f-h) of the boxed regions (a and e, respectively) (scale bar 0.15 mm). **B.** Visualization by confocal microscopy of intracellular neutral lipids (stained in green with BODIPY) in fat body cells of wild type and Dredd^D55^ mutant flies in control conditions (a, b and c) and infected with *Ecc* (d, e and f) for 10 or 20 days. (scale bar 20 μm). **C.** BODIPY staining of lipid droplets in fat body cells of control flies (UAS-IMD) (a) and those overexpressing IMD (Da^Gal4^>UAS-IMD, Tub^Gal80ts^) for 10 days. (scale bar 20 μm). In all confocal images, co-DAPI staining (in blue) was used to visualize the nuclei of fat body cells.

**Sup Figure 3. Phenotypes associated with chronic activation of NF-κB cascade by bacteria-derived peptidoglycan.** **A.** Representative pictures of the head and proboscis of wild type flies in control conditions (a) and upon enteric dysbiosis for 30 days (b) (scale bar, 100 μm). In all cases, the constitutive extension of the proboscis is associated with the abdominal bloating of flies chronically infected. **B.** Representative fluorescent pictures of DAPI stained egg chambers at stage 9-10 of oogenesis of wild type control (a) and chronically infected flies for 30 days (b). The healthy egg chamber shows dispersed chromatin in nurse cell nuclei (a) while the egg chamber of chronically infected animals is characterized by the presence of pyknotic and fragmented nuclei (b, red stars) (scale bar, 100 μm)

**Sup Figure 4. Expression profile of Gal4 drivers in the intestine, fat body and Malpighian tubule cells. A-G** Representative confocal images of the intestine (a), the fat body (b) and the Malpighian tubules (c) of transgenic flies expressin*g* the membrane tagged red fluorescent protein (mCD8-RFP) under the control of Mex^Gal4^ (A), R4^Gal4^ (B), C42^Gal4^ (C), Uro^Gal4^ (D), LKR^Gal4^ (E), Tret1-1^Gal4^ (F) and of Da^Gal4^ (G) drivers, respectively. c’, magnified Malpighian tubules views of the corresponding boxed regions (c). SC and PC stand for stellate and principal cells respectively.

**Sup Figure 5. Specific overexpression of PGRP-LCa in Malpighian tubule cells reduces life span and causes fluid buildup and fat body wasting. A.** Survival of control flies (UAS-PGRP-LCa, Tub^Gal80ts^) and flies overexpressing PGRP-LCa in enterocytes (Mex^gal4^>UAS-PGRP-LCa, Tub^Gal80ts^), fat body cells (R4^gal4^>UAS-PGRP-LCa, Tub^Gal80ts^) and in Malpighian principal (C42^gal4^>UAS-PGRP-LCa, Tub^Gal80ts^) or stellate cells (LKR^Gal4^>UAS-PGRP-LCa, Tub^Gal80ts^). The difference between control flies and those overexpressing PGRP-LCa is significant (p<0.001 respectively; one-sided log rank test). **B.** Representative pictures of control flies (a) and those overexpressing PGRP-LCa (b-e) (scale bar, 0.5 mm). a’-e’, magnified fat bodies views of the boxed regions (scale bar, 0.15 mm). a”-e”, representative pictures of ovaries from control flies and upon activation of the IMD cascade by overexpression of PGRP-LCa (scale bar, 0.5 mm)*.* **C-F.** Quantification of abdominal bloating (C), body weight (D), ovary atrophy (E) and ovarioles that contained apoptotic nurse cells (F) in control flies and those overexpressing PGRP-LCa. Comparisons between selected conditions are shown (Fisher’s exact test (C and E) and Mann Whitney tests (D and F); ns, not significant, **, p<0.01 and ***, p<0.001).

**Sup Figure 6. Overexpression of IMD in Malpighian tubule cells reduces life span and causes fluid retention and fat body degeneration. A.** Survival of control flies (UAS-IMD, Tub^Gal80ts^) and flies overexpressing IMD in Malpighian principal cells (Uro^gal4^>UAS-IMD, Tub^Gal80ts^) or stellate cells (Tret1-1^Gal4^>UAS-IMD, Tub^Gal80ts^). The difference between control flies and those overexpressing IMD is significant (p<0.001 respectively; one-sided log rank test). **B.** Representative pictures of control flies (a) and those overexpressing IMD (b and c) (scale bar, 0.5 mm). a’-c’, magnified fat bodies views of the boxed regions (scale bar, 0.15 mm). a”-c”, representative pictures of ovaries from control flies and upon activation of the IMD cascade by overexpression of IMD (scale bar, 0.5 mm). **C-F.** Quantification of abdominal bloating (C), body weight (D), ovary atrophy (E) and ovarioles that contained apoptotic nurse cells (F) in control flies and those overexpressing IMD. Comparisons between selected conditions are shown (Fisher’s exact test (C and E) and Mann Whitney tests (D and F); ns, not significant, **, p<0.01 and ***, p<0.001).

**Sup Figure 7. Simultaneous activation of the IMD cascade in principal and stellate cells impacts life span and induces early fluid retention and fat body wasting. A.** Quantitative RT-PCR analysis of the expression of the NF-κB target gene Diptericin in Malpighian tubules of flies overexpressing IMD in principal cells, stellate cells or in both cell types (C42^gal4^>UAS-IMD, Tub^Gal80ts^, LKR^Gal4^>UAS-IMD, Tub^Gal80ts^ and C42^gal4^; LKR^Gal4^>UAS-IMD, Tub^Gal80ts^ respectively). Results are presented relative to those of control flies (UAS-IMD, Tub^Gal80ts^). **B.** Survival of control flies (UAS-IMD, Tub^Gal80ts^) and flies overexpressing IMD in principal cells, stellate cells or in both cell types. The difference between flies overexpressing IMD simultaneously in principal and stellate cells and flies overexpressing IMD specifically one cell type is significant (p<0.001 respectively; one-sided log rank test). **C.** Representative pictures of control flies (a) and those overexpressing IMD (b-d) (scale bar, 0.5 mm). a’-d’, magnified fat bodies views of the boxed regions (scale bar, 0.15 mm). a”-d”, representative pictures of ovaries from control flies (a”) and upon activation of the IMD cascade by overexpression of IMD (b”-d”) (scale bar, 0.5 mm). **D-G.** Quantification of abdominal bloating, (D), body weight (E), ovary atrophy (F) and ovarioles that contained apoptotic nurse cells (G) in control flies and those overexpressing IMD. Comparisons between selected conditions are shown (Fisher’s exact test (C and E) and Mann Whitney tests (D and F); ns, not significant, **, p<0.01 and ***, p<0.001).

**Sup Figure 8. Overexpression of the cytosolic amidase PGRP-LB^RD^ in MT principal cells increases life span and prevents abdominal bloating during enteric dysbiosis.** **A.** Survival of control flies (UAS-PGRP-LB^RD^) and those overexpressing PGRP-LB^RD^ in MT principal cells (C42^Gal4^>UAS-PGRP- LB^RD^) upon chronic enteric dysbiosis. The difference between these conditions is significant (p<0.001; one-sided log rank test). **B-D.** Quantification of abdominal bloating (B), ovary degeneration (C) and ovarioles that contained apoptotic nurse cells (D) upon chronic infection in control flies and those overexpressing PGRP- LB^RD^ in principal cells. Comparisons between selected conditions are shown (Fisher’s exact test (B and C) and Mann Whitney test (D); ns, not significant, ***, p<0.001).

**Sup Figure 9.** **Modulation of the IMD signaling cascade in MT cells prevents insulin signaling inhibition during chronic enteric dysbiosis.** **A** and **B.** Quantitative RT-PCR analysis of 4EBP expression in Malpighian tubules of control and chronically infected flies for 1 and 20 days and after RNAi-mediated inactivation of Fadd (A) or overexpression of PGRP-LB^RD^ (B) in principal cells. Comparisons between selected conditions are shown (Fisher’s exact test; ns, not significant; *** P < 0.001, ** P < 0.01, * P < 0.1).
