# Supplementary material for: Gut bacteria-derived peptidoglycan induces a metabolic syndrome-like phenotype via NF-κB-dependent insulin/PI3K signaling reduction in Drosophila renal system
Source: Sci Rep. 2020 Aug 24;10:14097. doi: 10.1038/s41598-020-70455-7 (PMC7445169; doi:10.1038/s41598-020-70455-7)
Supplement: Supplementary file 2 — Supplementary Information 2. [file 41598_2020_70455_MOESM2_ESM.pdf]

Supplementary figures

Gut bacteria-derived peptidoglycan induces a metabolic syndrome-like phenotype via NF-κB-dependent insulin/PI3K signaling reduction in *Drosophila* renal system

Olivier Zugasti, Raphäel Tavignot, Julien Royet

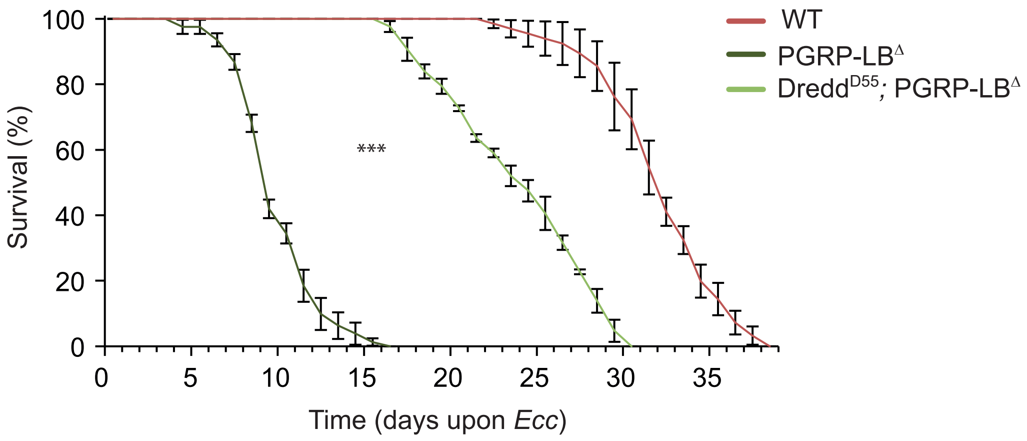

100
80
60
40
20
0
Survival (%)
0
5
10
15
20
25
30
35
Time (days upon Ecc)
***
WT
PGRP-LBΔ
DreddD55; PGRP-LBΔ

Sup. Fig 1

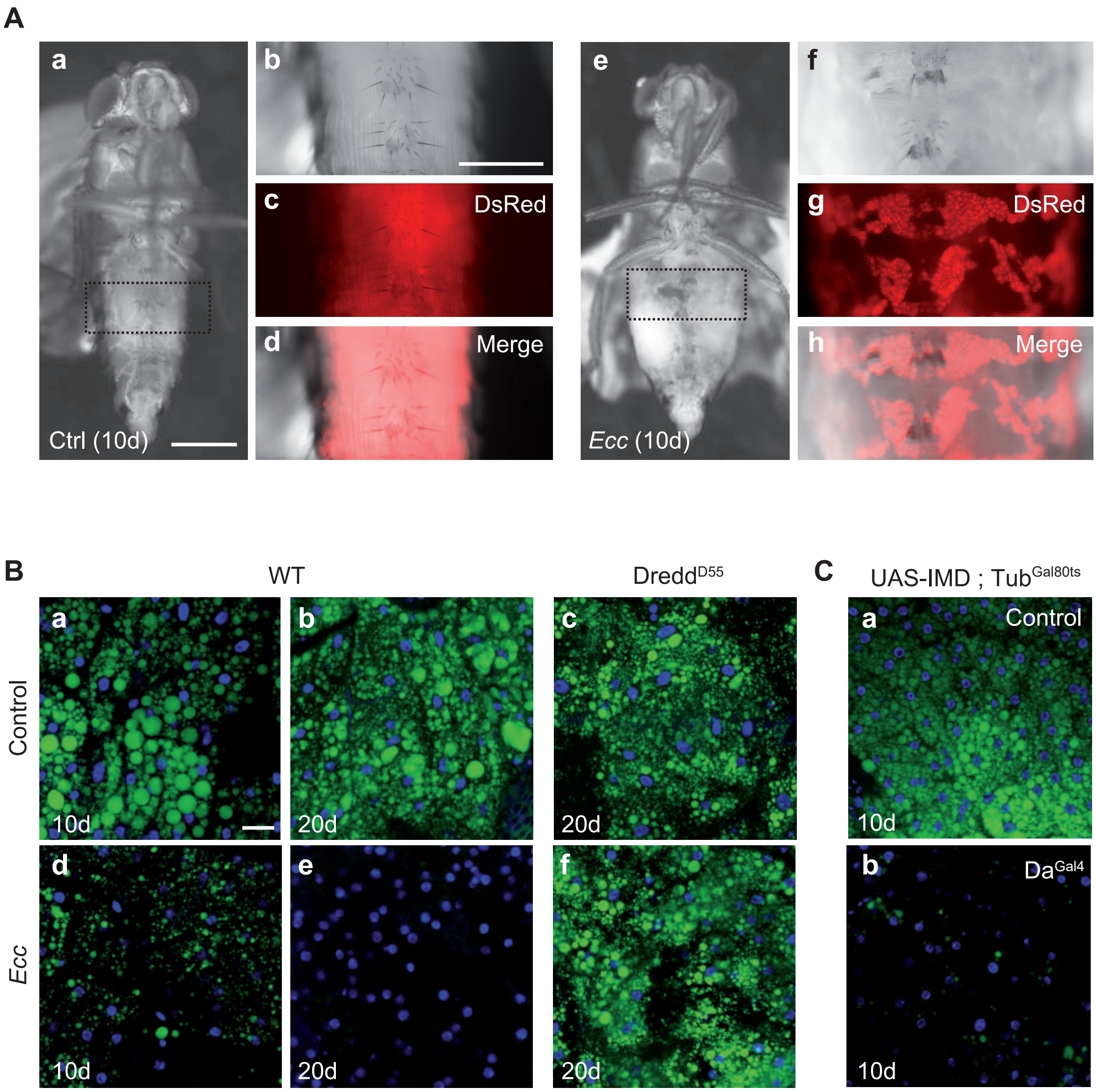
A
a
Ctrl (10d)
b
c
DsRed
d
Merge
e
Ecc (10d)
f
g
DsRed
h
Merge
B
WT
DreddD55
Control
Ecc
a
10d
b
20d
c
20d
d
10d
e
20d
f
20d
C
UAS-IMD ; TubGal80ts
a
Control
10d
b
DaGal4
10d

Sup. Fig 2

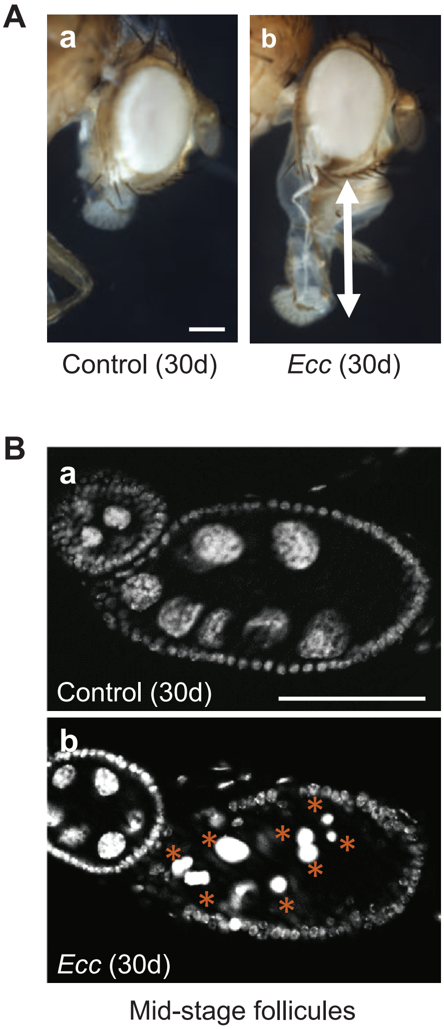
A
a
b
Control (30d)
Ecc (30d)
B
a
Control (30d)
b
Ecc (30d)
Mid-stage follicules

Sup. Fig 3

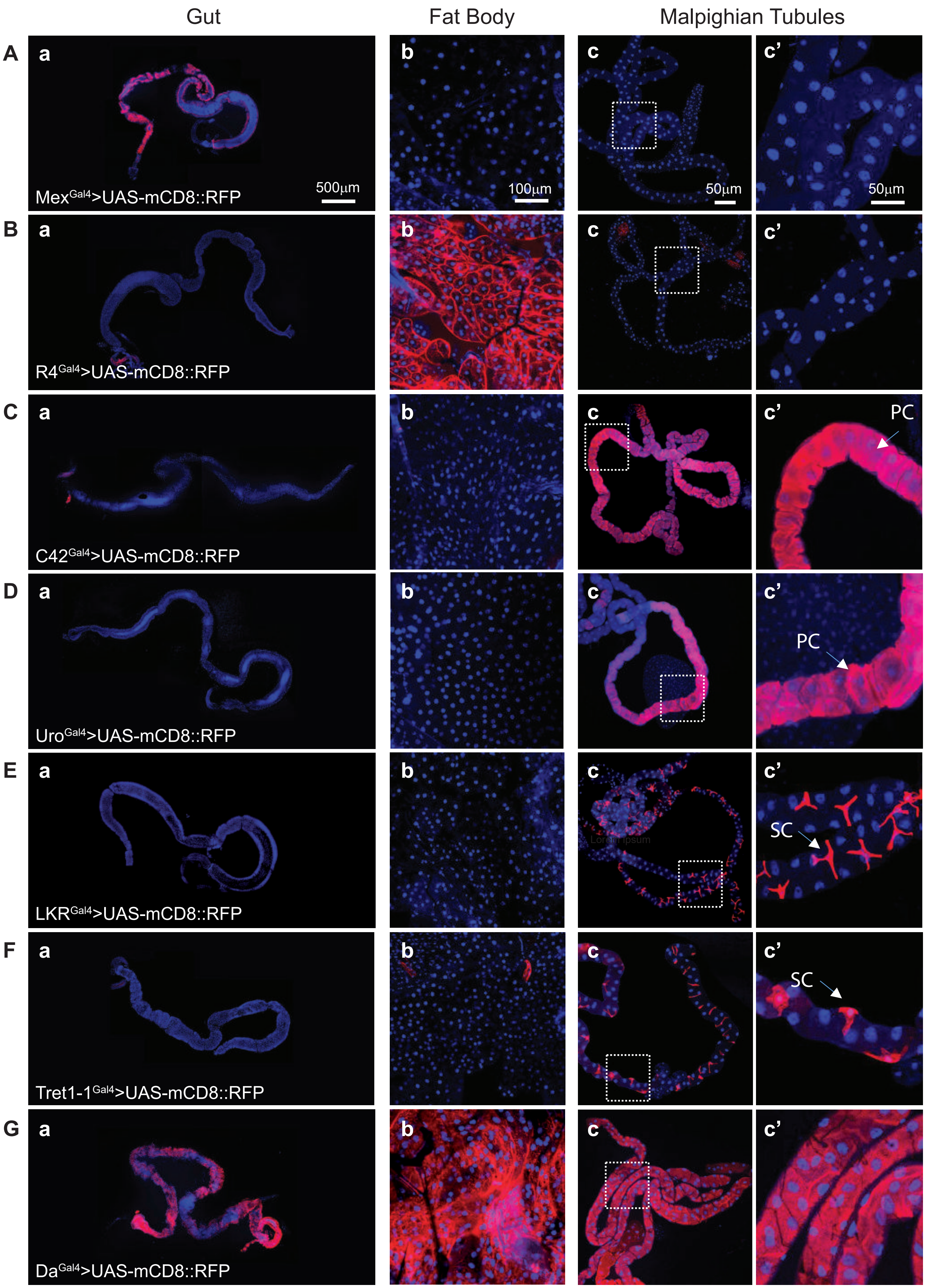
Gut
Fat Body
Malpighian Tubules
A
a
b
c
c’
Mex^Gal4>UAS-mCD8::RFP
500µm
100µm
50µm
50µm
B
R4^Gal4>UAS-mCD8::RFP
C
C42^Gal4>UAS-mCD8::RFP
PC
D
Uro^Gal4>UAS-mCD8::RFP
PC
E
LKR^Gal4>UAS-mCD8::RFP
SC
F
Tret1-1^Gal4>UAS-mCD8::RFP
SC
G
Da^Gal4>UAS-mCD8::RFP

Sup. Fig 4

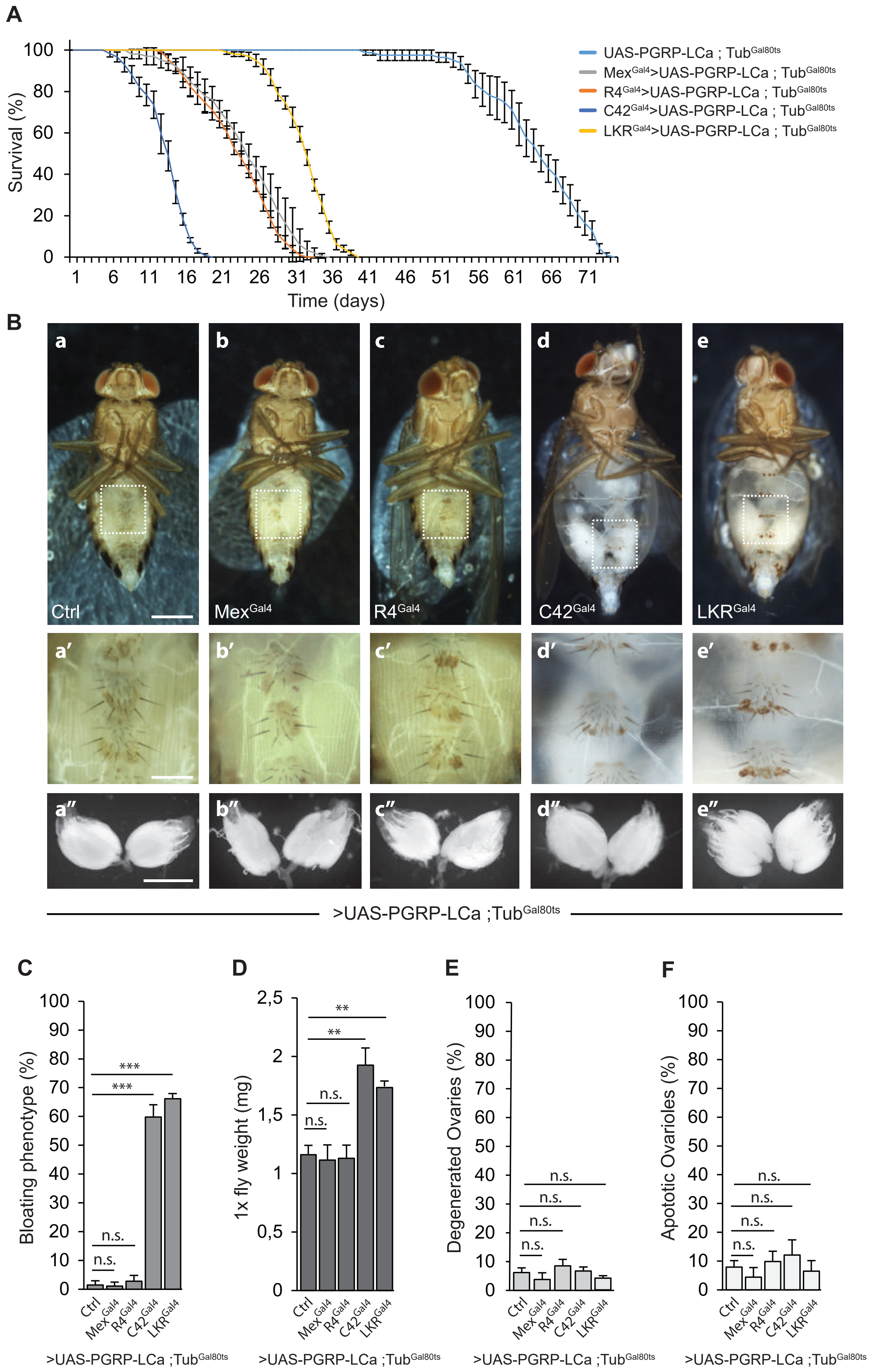
A
Survival (%)
Time (days)
UAS-PGRP-LCa ; Tub^Gal80ts
Mex^Gal4>UAS-PGRP-LCa ; Tub^Gal80ts
R4^Gal4>UAS-PGRP-LCa ; Tub^Gal80ts
C42^Gal4>UAS-PGRP-LCa ; Tub^Gal80ts
LKR^Gal4>UAS-PGRP-LCa ; Tub^Gal80ts
B
Ctrl
Mex^Gal4
R4^Gal4
C42^Gal4
LKR^Gal4
>UAS-PGRP-LCa ;Tub^Gal80ts
C
Bloating phenotype (%)
D
1x fly weight (mg)
E
Degenerated Ovaries (%)
F
Apototic Ovarioles (%)
n.s.
***
**

Sup. Fig 5

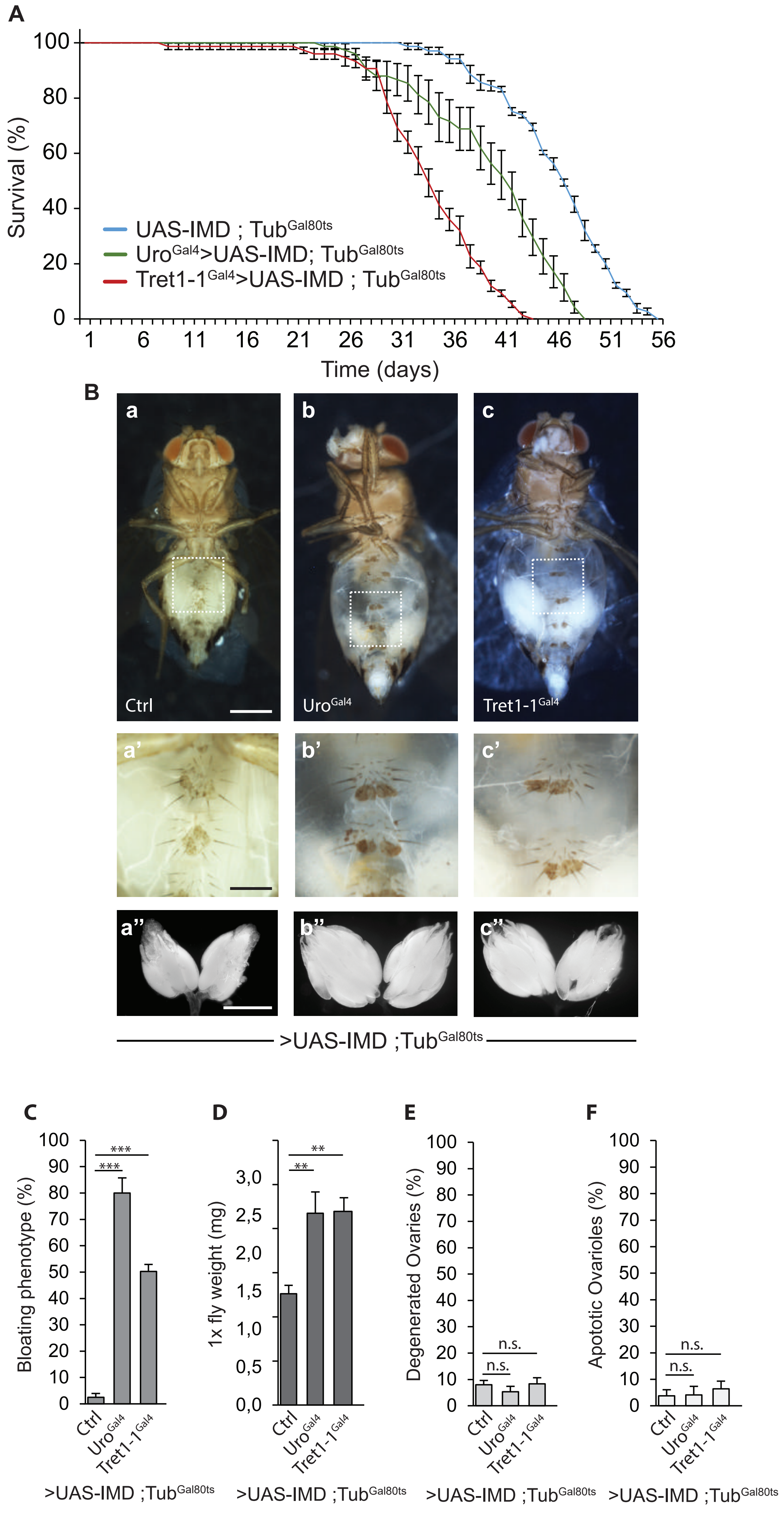

A
100
80
60
40
20
0
Survival (%)
UAS-IMD ; Tub^Gal80ts
Uro^Gal4>UAS-IMD; Tub^Gal80ts
Tret1-1^Gal4>UAS-IMD ; Tub^Gal80ts
1
6
11
16
21
26
31
36
41
46
51
56
Time (days)
B
a
b
c
Ctrl
Uro^Gal4
Tret1-1^Gal4
a'
b'
c'
a''
b''
c''
>UAS-IMD ;Tub^Gal80ts
C
D
E
F
Bloating phenotype (%)
1x fly weight (mg)
Degenerated Ovaries (%)
Apototic Ovarioles (%)
***
**
n.s.
Ctrl
Uro^Gal4
Tret1-1^Gal4
>UAS-IMD ;Tub^Gal80ts

Sup. Fig 6

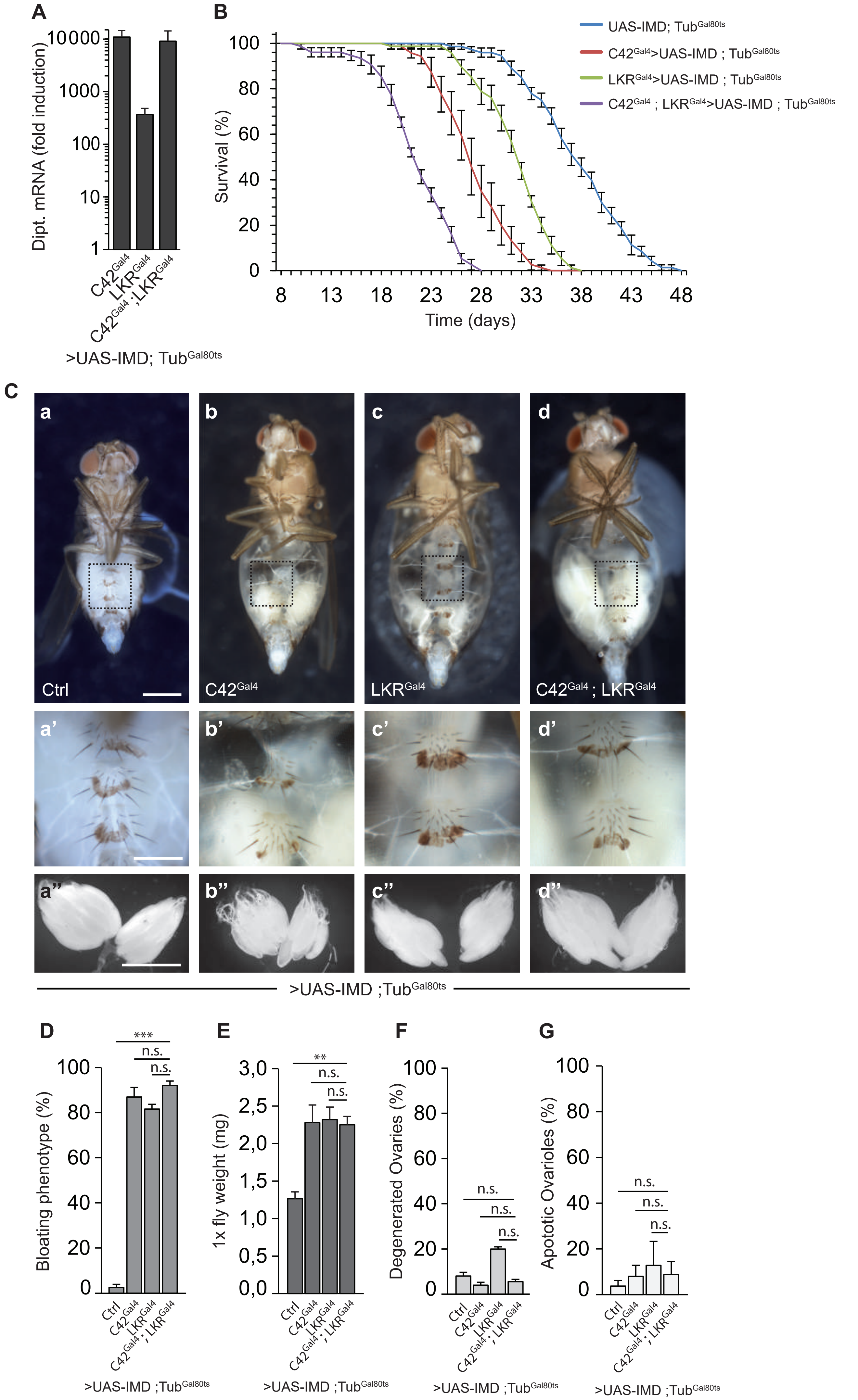
A
B
Dipt. mRNA (fold induction)
10000
1000
100
10
1
C42Gal4
LKRGal4
C42Gal4;LKRGal4
>UAS-IMD; TubGal80ts
Survival (%)
100
80
60
40
20
0
8
13
18
23
28
33
38
43
48
Time (days)
UAS-IMD; TubGal80ts
C42Gal4>UAS-IMD ; TubGal80ts
LKRGal4>UAS-IMD ; TubGal80ts
C42Gal4 ; LKRGal4>UAS-IMD ; TubGal80ts
C
a
b
c
d
Ctrl
C42Gal4
LKRGal4
C42Gal4 ; LKRGal4
a'
b'
c'
d'
a''
b''
c''
d''
>UAS-IMD ;TubGal80ts
D
E
F
G
***
n.s.
Bloating phenotype (%)
**
1x fly weight (mg)
3,0
2,5
2,0
1,5
1,0
0,5
0,0
Degenerated Ovaries (%)
Apotoic Ovarioles (%)
Ctrl
C42Gal4
LKRGal4
C42Gal4 ; LKRGal4
>UAS-IMD ;TubGal80ts

Sup. Fig 7

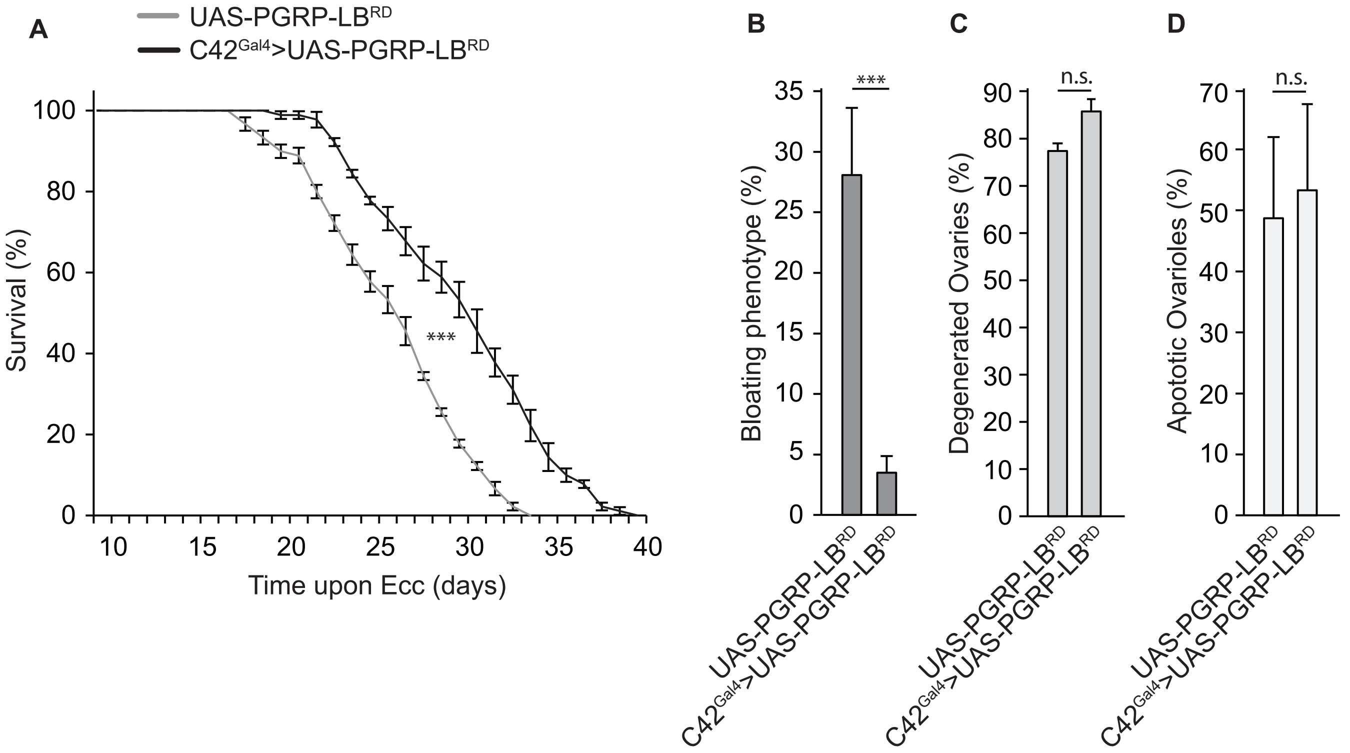
A
UAS-PGRP-LBRD
C42Gal4>UAS-PGRP-LBRD
Survival (%)
Time upon Ecc (days)
***
B
Bloating phenotype (%)
***
C
Degenerated Ovaries (%)
n.s.
D
Apototic Ovarioles (%)
n.s.
UAS-PGRP-LBRD
C42Gal4>UAS-PGRP-LBRD

Sup. Fig 8

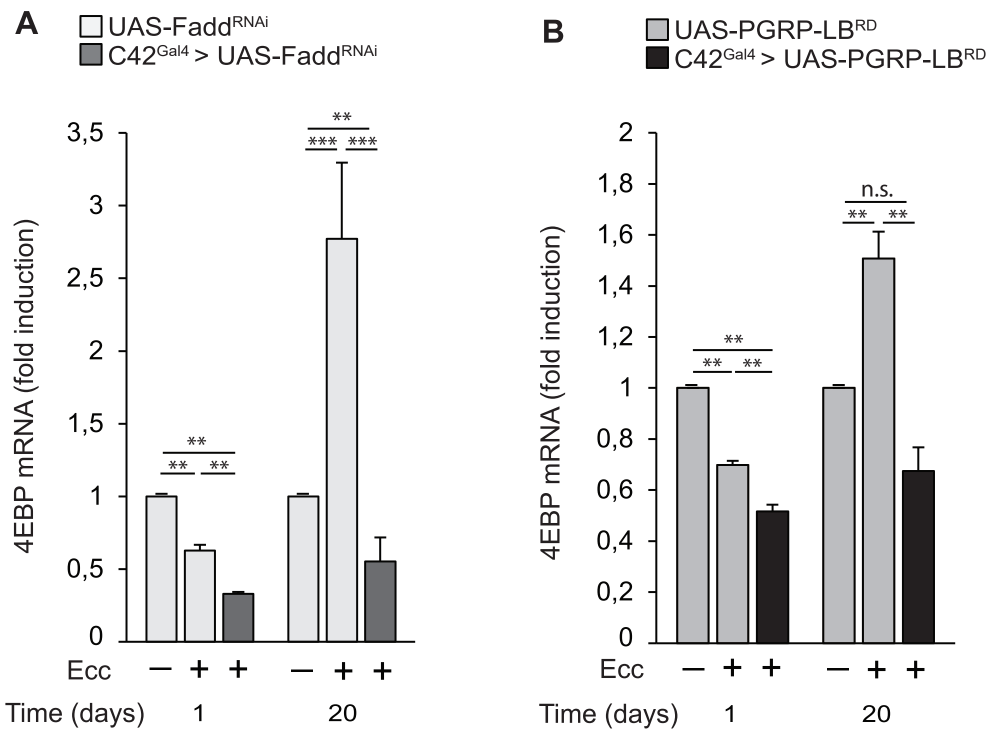
A
UAS-Fadd RNAi
C42 Gal4 > UAS-Fadd RNAi
4EBP mRNA (fold induction)
3,5
3
2,5
2
1,5
1
0,5
0
**
***
Ecc
— + + — + +
Time (days)
1
20
B
UAS-PGRP-LB RD
C42 Gal4 > UAS-PGRP-LB RD
4EBP mRNA (fold induction)
2
1,8
1,6
1,4
1,2
1
0,8
0,6
0,4
0,2
0
n.s.
**
Ecc
— + + — + +
Time (days)
1
20

Sup. Fig 9
